# An atypical presentation of a mesenteric Meckel’s diverticulum in a 7-year-old warmblood mare: case report

**DOI:** 10.1186/s12917-020-02631-w

**Published:** 2020-10-29

**Authors:** E. Stas, L. Kranenburg, P. Witt, J. de Grauw, J. van den Brand, J. Ensink, H. Brommer

**Affiliations:** 1 Department Clinical Sciences, Utrecht University, Faculty of Veterinary Medicine, Yalelaan 112, 3584 CM Utrecht, the Netherlands; 2 Division of Pathology, Utrecht University, Faculty of Veterinary Medicine, Yalelaan 1, 3584 CM Utrecht, the Netherlands

**Keywords:** Meckel’s diverticulum, Equine, Strangulation, Colic, Case report

## Abstract

**Background:**

Meckel’s diverticula are a rare cause of small intestinal strangulation, diagnosed at laparotomy or necropsy. This congenital anomaly of the gastrointestinal tract originates from a remnant of the vitelline duct. In reported equine cases, they present as a full-thickness diverticulum on the antimesenteric border of the distal jejunum or proximal ileum.

**Case presentation:**

On laparotomy a Meckel’s diverticulum positioned at the mesenteric side was found to be the cause of small intestinal strangulation. This position is very uncommon and to the best knowledge of the authors there is no unambiguous description of another case.

**Conclusions:**

Meckel’s diverticula should be on the list of differential diagnoses in cases of small intestinal strangulation. As in humans, equine Meckel’s diverticula can have the standard antimesenteric as well as a more exceptional mesenteric location. This case adds to the series of anecdotal reports of anomalies with regard to Meckel’s diverticula in the horse.

## Background

Vitelline anomalies are a rare and often overlooked cause of small intestinal strangulation in horses [1, 2]. During the early embryonic stages, the vitelline duct (also called omphalomesenteric duct) connects the yolk sac with the developing midgut [3–5]. As the yolk regresses and the placenta takes over, the vitelline duct and paired vitelline arteries atrophy [3, 5]. In case of an incomplete obliteration, a mesodiverticular band, a vitelline duct cyst or a Meckel’s diverticulum may remain [2, 3, 5–8]. Vitelline remnants can be an incidental finding during exploratory laparotomy, but they may as well lead to small intestinal strangulation for which surgical intervention is needed [1, 3, 8].

This report describes the case of a 7-year old warmblood mare with an atypical mesenteric positioned Meckel’s diverticulum as the cause of small intestinal strangulation diagnosed at exploratory laparotomy.

## Case presentation

### History

A 7-year-old, 495 kg Dutch warmblood mare developed signs of acute colic (attempts to lie down, rolling, restlessness and pawing) after riding. The horse did not respond to intravenous analgesia with flunixin meglumine given by the referring veterinarian and was referred to the Utrecht University Equine Clinic (Utrecht, the Netherlands). The mare arrived at the university clinic 2.5 h after the onset of symptoms.

### Clinical examination

On presentation, the mare continued showing signs of abdominal pain. A general physical examination was performed, which revealed a heart rate of 48 beats/min, a respiratory rate of 10 breaths/min and a rectal temperature of 37.2 °C. Oral mucous membranes were slightly pale and tacky with a prolonged CRT. The abdomen was slightly distended. On abdominal auscultation borborygmi were absent on the left and reduced in frequency on the right side. Upon nasogastric intubation no reflux was obtained. Rectal examination revealed a mildly impacted pelvic flexure, no distended loops of small intestine could be palpated or seen on transcutaneous abdominal ultrasonography at this timepoint. Results of a hematological assessment and biochemical panel were still within normal limits.

Based on these clinical findings and particularly on the severe persisting signs of colic, which were unresponsive to repeated analgesia and sedation, a tentative clinical diagnosis of a small intestinal strangulation was made. The mildly impacted pelvic flexure was assumed to be secondary. An exploratory laparotomy under general anesthesia was recommended.

### Surgery

Pre-operatively the horse received benzylpenicillin (Benzyl-G 10Mega^A^) at 20.000 IU/kg body weight (BW) intravenously (IV) and gentamycin (Gentamycine 5%^B^) at 6.6 mg/kg BW IV. The horse was premedicated with 1 ml of detomidine (Domosedan 10 mg/ml^C^) IV and morphine (Morfine 10 mg/ml^D^) at 0.1 mg/kg BW IV, induced with ketamine (Narketan 100 mg/ml^E^) at 2.2 mg/kg BW IV and midazolam (Midazolam Aurobindo 5 mg/ml^F^) at 0.06 mg/kg BW IV. Anesthesia was maintained with isoflurane (IsoFlo^G^) in oxygen. At the end of the surgery procaine penicillin (Procapen 300 mg/ml^H^) at 20.000 IU/kg BW was injected intramuscularly (IM).

**Fig. 1:**
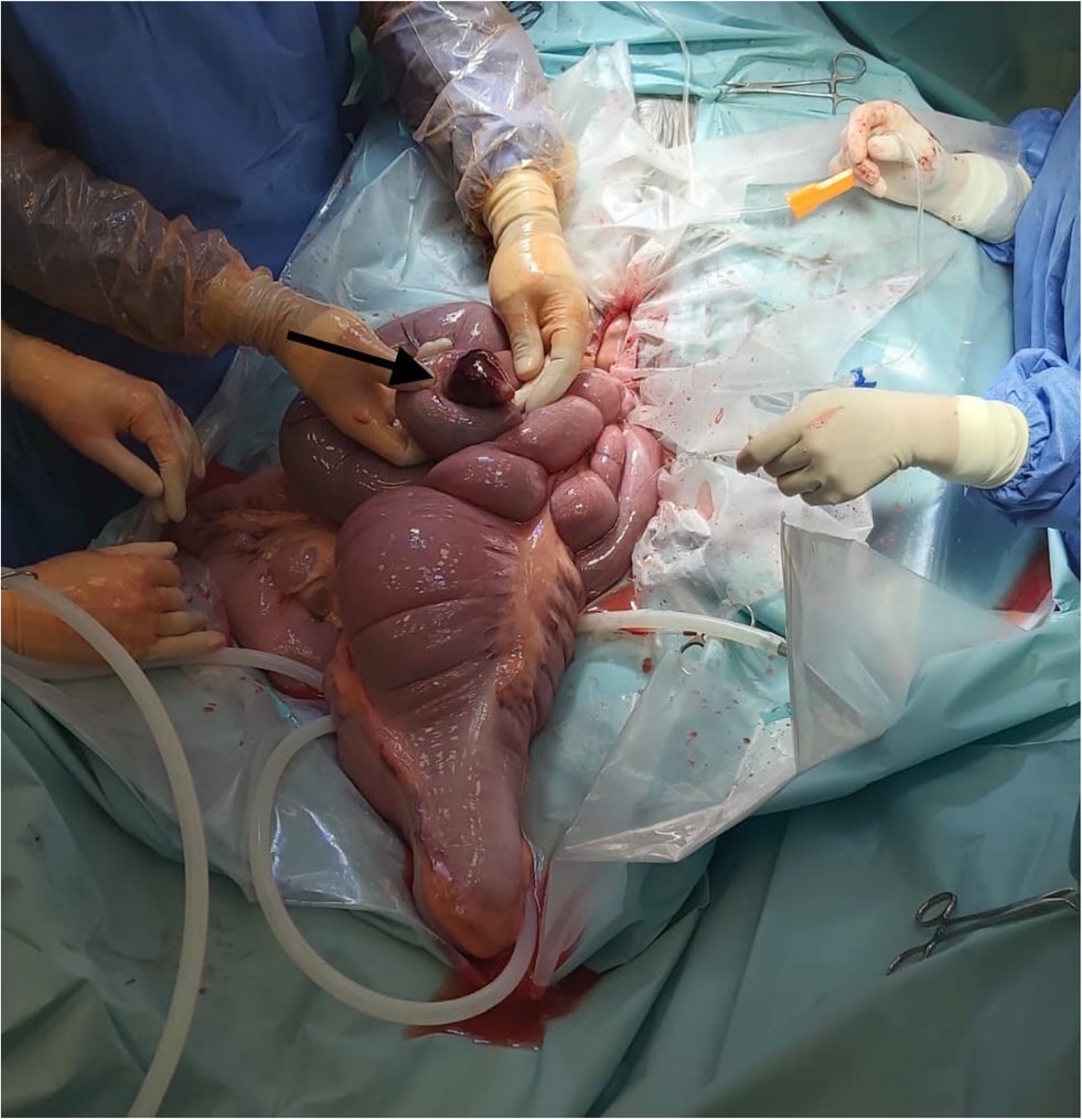
Intra-operative view of the strangulation in the distal jejunum. Arrow: The distal tip of the diverticulum, a hemorrhagic fat pedicle, secured the loop of strangulation. Note the surrounding dilated small intestines

**Fig. 2:**
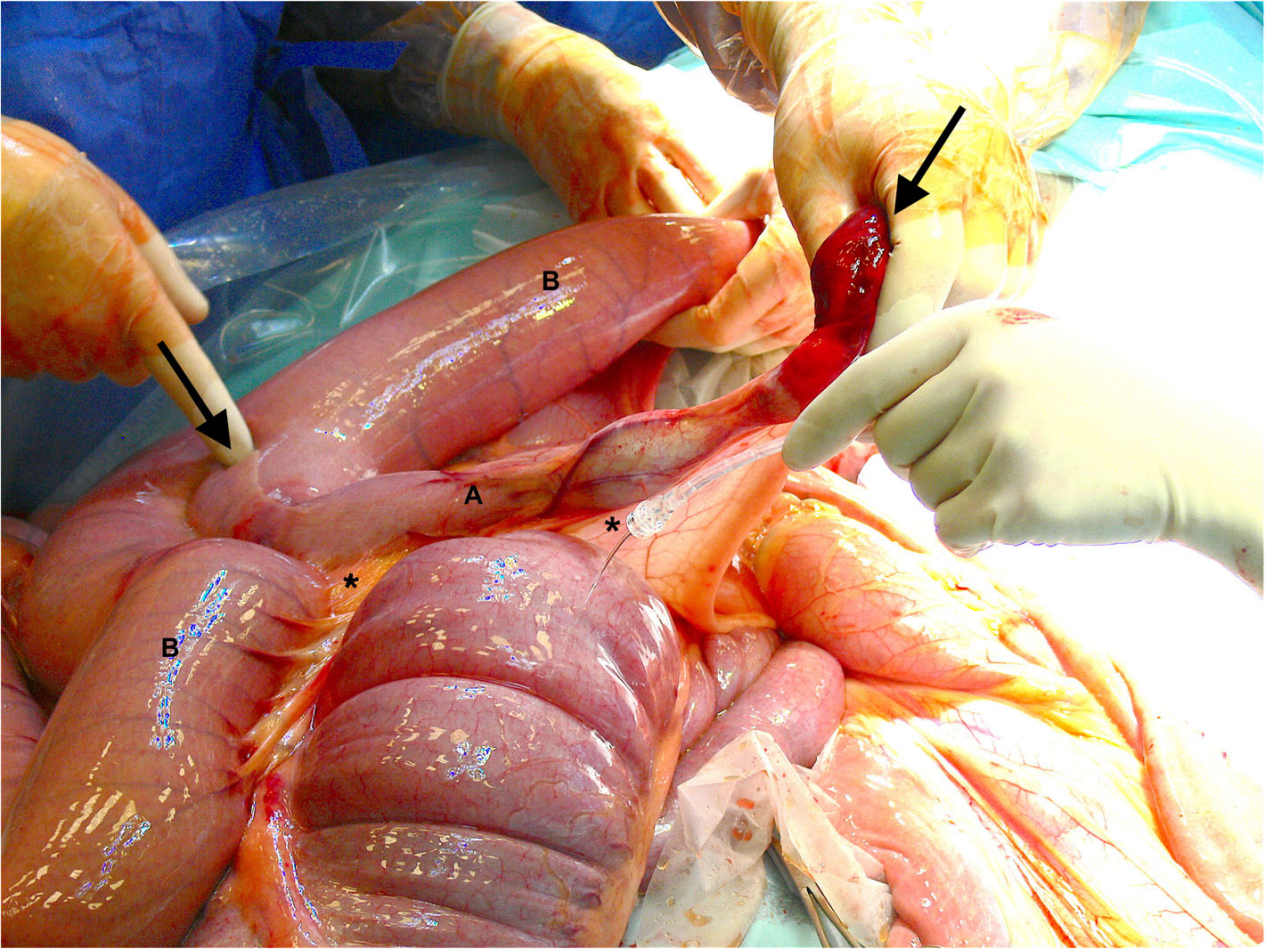
Intra-operative view of the Meckel’s diverticulum (**a**) and jejunum (**b**) after reduction of the strangulation. The mesenteric (*) positioned diverticulum (between the black arrows) first runs parallel to the jejunum and diverges after 17 cm into a free diverticulum

With the horse in dorsal recumbency a standard midline approach was used, and a 25 cm incision was made through the linea alba. Manual exploration of the abdomen revealed a small intestinal strangulation, several dilated loops of small intestine and a mild impaction of the ascending colon. The strangulation was located in the distal jejunum (Figure 1), which after reduction and reposition appeared to have a Y-shaped anatomy in this horse (Figure 2). Given its appearance a congenital anomaly was presumed. The tube-like diverticulum originated from the mesenteric side of the jejunum instead of the anti-mesenteric side, as it is most often described for a Meckel’s diverticulum [9–11]. Oral to the strangulation, the jejunum was fluid filled and distended for a length of 6–7 m. After correction of the strangulation, the small intestines were decompressed by gently pushing its contents into the cecum before resection was started.

**Fig. 3:**
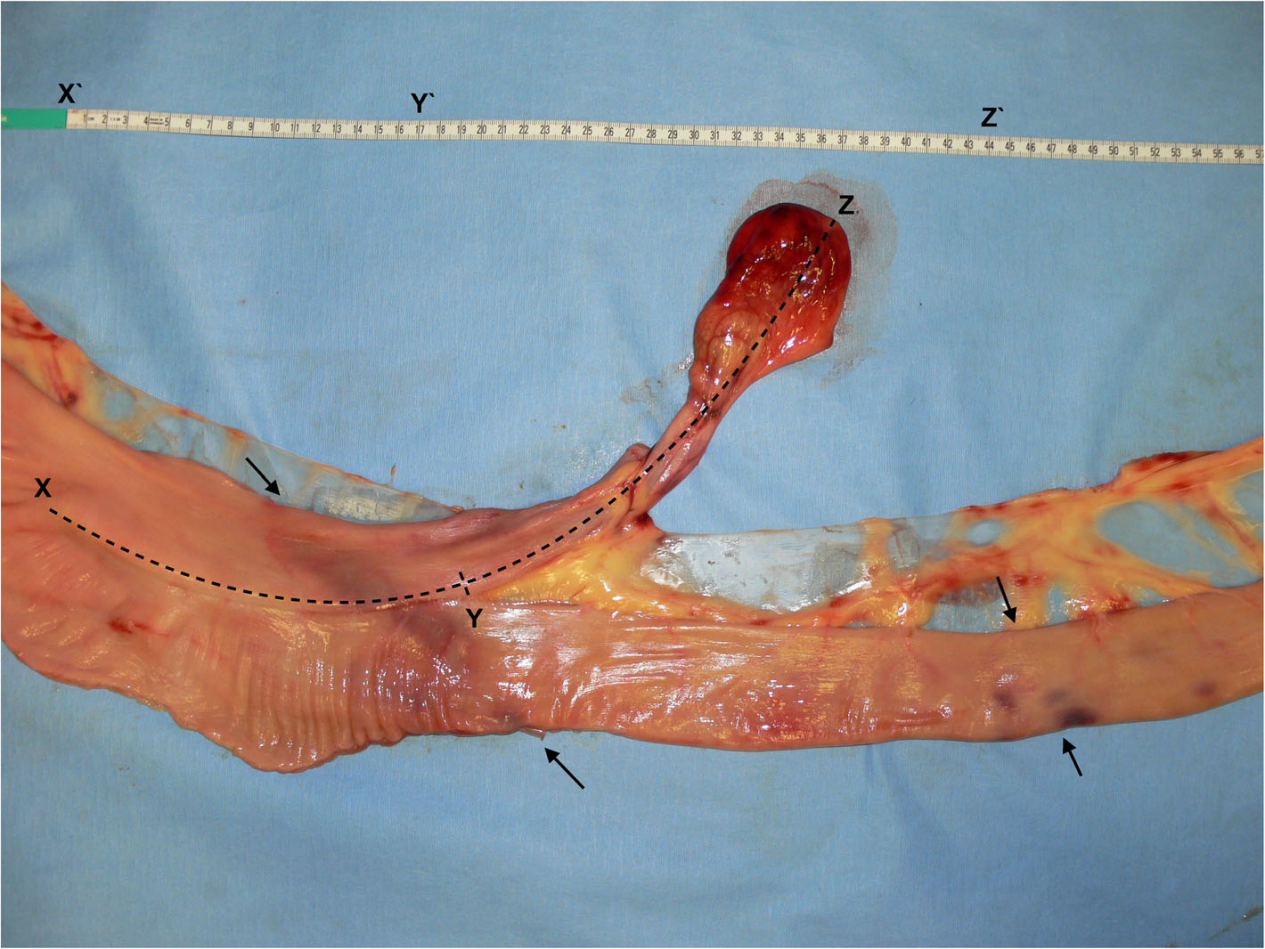
Measurements of the diverticulum. Left is proximal and right is distal. Measuring 44 cm in full length (X-Z); 17 cm parallel to the jejunum (X-Y), with a free part of 27 cm (Y-Z). Black arrows point out the limits of strangulation; a 30 cm loop of jejunum was strangulated by the diverticulum

The blind-ending diverticulum was located in the distal segment of the jejunum not involving the jejuno-ileal junction, it measured 44 cm in total length and 5 cm in diameter. The proximal 17 cm ran parallel to the jejunum after which it diverged to a free diverticulum (27 cm) with its own mesentery (Figure 3). The luminal part had a full length of 34,5 cm. The distal tip, a fat pedicle that secured the loop of strangulation (Fig. 1), had a dark red hemorrhagic serosal discoloration. The serosa of the adjacent jejunum showed a band of pink-purple discoloration on the sides of strangulation; in total, a loop of 30 cm of jejunum was strangulated by the diverticulum (Fig. 3).

Intestinal motility appeared to be decreased orally and normal aborally of the strangulation. All parts of intestine were considered viable based on their motility, wall thickness and color. However, to prevent reoccurrence of a strangulation or other complications caused by the diverticulum (e.g. impaction), the decision was made to perform ‘en bloc’ resection of the anomaly and the associated jejunum of approximately 1 m. An end-to-end jejuno-jejunostomy single-layer anastomosis as described by Freeman (2019) [8] was performed. Vessels in the mesentery were ligated with polyglactin 910 (Vicryl^I^) USP 0. The one-layered anastomosis was created with an interrupted Lembert pattern of polydioxanone (PDS^J^) USP 3–0. The mesentery was closed with a simple continuous suture of polyglactin 910 (Vicryl^I^) USP 0. At the end of surgery 1 L of carboxymethyl cellulose (Carmellose gel 2%^K^) was deposited intra-abdominally and the horse received dalteparin (Fragmin^L^, 50 IU/kg) subcutaneously to prevent intestinal adhesions. The abdominal wall was closed in four layers: the peritoneum was sutured with a simple continuous suture pattern of polyglactin 910 (Vicryl^I^) USP 0, the linea alba with a simple continuous suture pattern of polyglactin 910 (Surgicryl^M^) USP 6, the subcutis with a simple continuous suture pattern of polyglactin 910 (Vicryl^I^) USP 2–0 and finally the skin with a continuous intradermal suture pattern of poliglecaprone (Monocryl^N^) USP 2–0. A rolled gauze stent was sutured over the wound with interrupted cruciate sutures of nylon (Ethylon^O^) USP 0. Recovery from anesthesia was uneventful.

### Histopathology

Histopathological examination of the resected intestine was performed. The lumen of the diverticulum was covered by a stratified squamous epithelium. The second layer consisted of loose fibrous tissue, followed by a layer of adipose tissue and finally at the periphery a mesothelial, serosa-like layer. There was no lamina muscularis present in the diverticulum. The histological examination of the neighboring jejunum showed that all expected anatomical layers were present.

### Follow-up

Following surgery, the horse was admitted to the intensive care ward with treatment consisting of continuous infusion of fluids (Ringer^P^, lidocaine; 50 mcg/kg/min), antibiotics (Gentamycin, Gentamycine 5%^B^; 6.6 mg/kg BW IV q24h and procainpenicillin, Procapen 300 mg/ml^H^; 20.000 IU/kg BW IM q24h) NSAIDs (Flunixine meglumine, Megluxin^R^; 1.1 mg/kg BW IV q12h), low-molecular weight heparin (Dalteparin, Fragmin^L^; 50 IU/kg BW SC q24h) and gastroprotectants (Omeprazole, Gastrogard^S^; 1 mg/kg BW PO q24h). The horse was regularly checked for reflux by nasogastric intubation. The first 24 h after surgery the horse was fasted and allowed to drink 2 L/2 h, after which silage and grass were slowly reintroduced.

In the early post-operative period, the horse developed post-operative ileus with enterogastric reflux and mild colic signs (decreased appetite, weight shifting and pawing). On rectal examination, the previously encountered mild colonic impaction had increased in size, consistency was much firmer as a result of dehydration. Medical treatment with laxatives and metoclopramid CRI (Emeprid^T^, 0.04 mg/kg BW/h) did not resolve the condition. Based on these findings, and as colic persisted, the decision was made to perform a re-laparotomy. During repeat laparotomy, the anastomosis was checked and assessed to be patent and viable, with no impacted ingesta and a normal luminal diameter. The small intestines were decompressed to the cecum and the impaction in the ascending colon was resolved through an enterotomy at the pelvic flexure.

Post-operative course after the second laparotomy was favorable. Supportive therapies were gradually discontinued based on clinical findings and bloodwork. Infusions and Dalteparin were stopped after 3 days. Antibiotics were discontinued after 5 days. Gastroprotectants and NSAIDs were given for 10 days after surgery; 5 days of intravenous flunixin meglumine (Megluxin^Q^) at 1.1 mg/kg BW q12h was followed by 5 days of oral meloxicam (Metacam^U^) at 0.6 mg/kg BW q24h. The amount of roughage and soaked feed was gradually increased until the horse was back to full ration 8 days after the second surgery. The horse was discharged after 14 days of hospitalization.

Four months after discharge the owner was contacted by telephone for follow-up. The mare had not shown any new episodes of colic, nor signs of wound infection during the previous months. Riding had been gradually reintroduced, at the time of the telephone survey the mare was participating in her first competition at its previous level.

## Discussion and conclusions

**Fig. 4:**
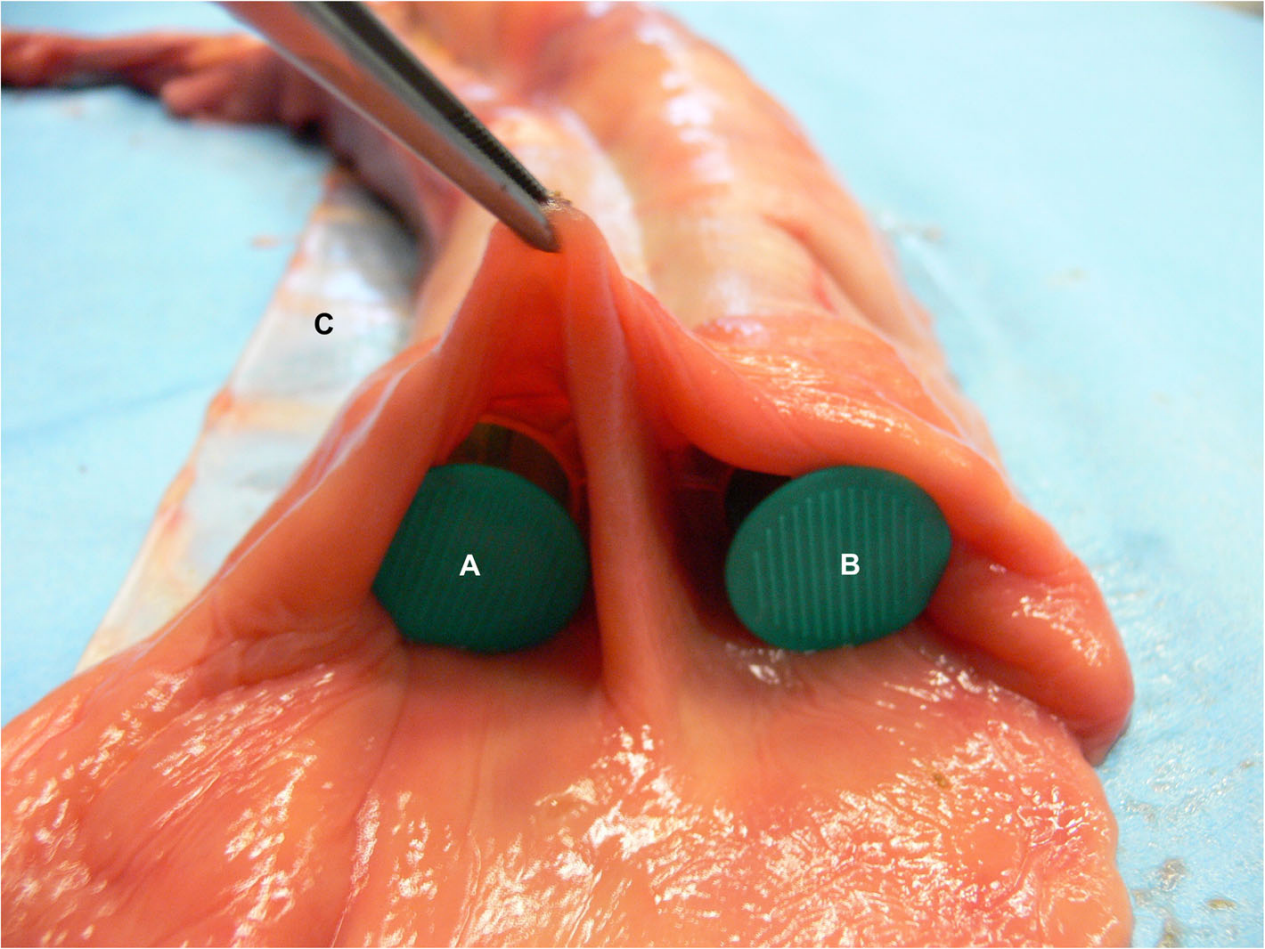
Bifurcation of the Meckel’s diverticulum (**a**) and jejunum (**b**); sharing the serosa but separated by a mucosa-covered septum. The diverticulum is positioned on the side of the mesenterium (**c**)

Meckel’s diverticula (MD) are a congenital disorder of the gastrointestinal tract found in several species like humans, pigs and horses [1, 2, 12]. In rare cases the vitelline duct (partially) fails to atrophy during embryonic development and a tube-like diverticulum remains, called a Meckel’s diverticulum [2, 3, 8]. This blind ending branch of small intestine is located at the distal end of the jejunum or proximal ileum, has a lumen and can vary in length from 10 to 35 cm [1, 3, 13]. The diverticulum encountered in this case consisted orally of a part that was attached to and ran parallel to the distal jejunum, sharing the serosa but separated by a mucosa-covered septum (Figure 4), similar to the cases described by Yovich and Horney (1983) [14] and Barakazai et al. (2003) [1]. Because of this, the diverticulum measured a total length of 44 cm, with a free part of 27 cm in length (Fig. 3). It has been postulated that the stratified squamous epithelium, as found in the diverticular part, might develop secondary to a chronic impaction [1, 2]. Similar to the findings described by Verwilghen et al. (2010) [2], no lamina muscularis could be identified in the case described here.

**Fig. 5:**
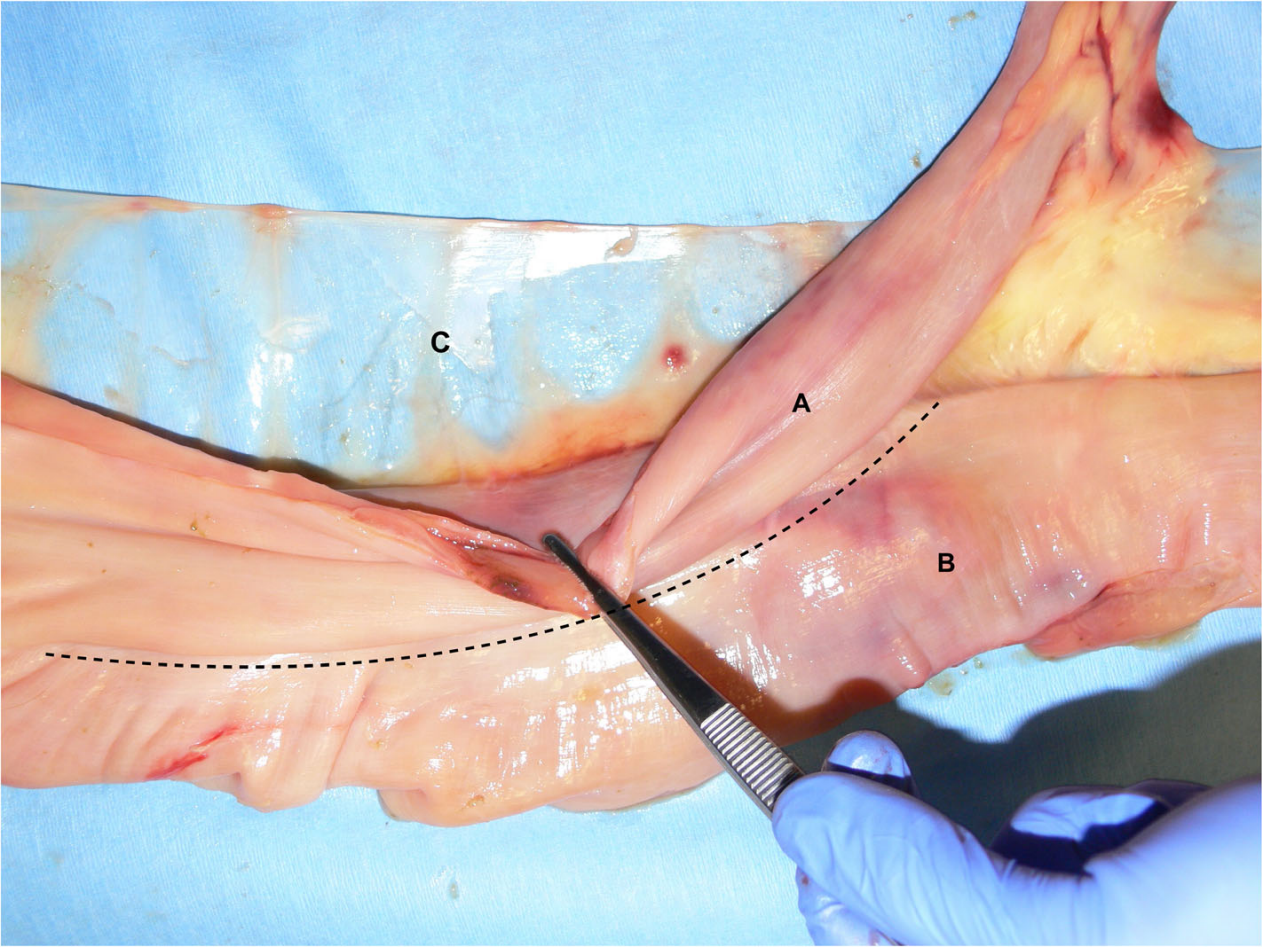
The Meckel’s diverticulum (**a**) in this case was atypically positioned on the mesenteric side (**c**) of the jejunum (**b**)

In literature a Meckel’s diverticulum is usually characterized by an origin at the antimesenteric aspect of the small intestine, however in humans there are a few cases known to have had a mesenteric positioned diverticulum [12, 15, 16]. To our knowledge this is the first equine case report to describe a mesenteric positioned tube-like diverticulum of the distal jejunum (Figure 5). The unusual condition encountered in this case is of value to report to assist surgeons encountering this during an emergency laparotomy as its location also influences the surgical possibilities. The case report by Wefel et al. (2011) [17] also describes a mesenteric diverticulum associated with a mesodiverticular band, however as that case did not present the typical anatomy of a blind conical extension of a Meckel’s diverticulum, it might have been an acquired diverticulum [17–19]. The anatomy of the diverticulum in the case described here must have been a congenital anomaly rather than an acquired one. Based on the typical anatomy, location in the distal jejunum and histological composition, this is very likely a variety of a Meckel’s diverticulum rather than a jejunal duplication [12] (Fig. 3).

Just like a Meckel’s diverticulum, a mesodiverticular band is a kind of vitelline anomaly [20]. A mesodiverticular band is a remnant of a vitelline artery, usually the left, and its associated mesentery [6, 8, 21]. This creates a triangular fold between the intestinal mesentery and attaching to the anti-mesenteric side of the distal jejunum [8, 10, 20]. These vitelline anomalies are rather rare findings: Van den Boom and Van der Velden (2001) [22] listed 5 out of 224 surgical cases of small intestinal strangulation to be caused by a mesodiverticular band (3) or Meckel’s diverticulum (2). Verwilghen et al. (2010) [2] reported that 2 out of 1008 horses that were admitted for acute colic had Meckel’s diverticula on laparotomy or post-mortem examination. Sprinkle et al. (1984) [3] found only 5 Meckel’s diverticula on 15,000 (0,03%) post-mortem examinations. Vitelline anomalies can go undetected and a horse can live without complications for years, as is reflected by the wide age range of cases reported in literature [1]. Based on this, the figures mentioned above might be an underestimation of the real prevalence in the equine population [9, 23].

Meckel’s diverticula can be an incidental finding; however, they are most often found to be related to the cause of colic [3, 8, 9]. They can cause intestinal strangulation, form an axis for volvulus, can be herniated or the blind ending can become impacted and ultimately rupture causing a fatal peritonitis [4, 8, 13, 18, 24, 25]. Similarly, mesodiverticular bands can lead to volvulus, small intestines can become strangulated in the blind sac or through a rent in the mesodiverticular band [8, 17, 20]. In the case described here the diverticulum was entangled around an adjacent part of distal jejunum, while the blind ending of the diverticulum was only slightly impacted with feed material.

Surgical intervention is straightforward for symptomatic diverticula, however a prophylactic surgical intervention for asymptomatic incidental diverticula is debatable [9, 12, 26]. Based on the risk factors, most surgeons advise a resection of the diverticulum or mesodiverticular band if found at laparotomy, as it may predispose for episodes of colic [11, 16, 17, 27]. In human medicine several surgical options have been described for cases of Meckel’s diverticula [27]. Based on the encountered anatomy, its relation to the colic signs and if applicable the viability of the strangulated part, a diverticulectomy, a wedge resection or a segmental resection is performed [25, 26]. Because of its location a diverticulectomy as described by Bartmann et al. (2002) [13] was not possible in this case as this would impair blood supply to the remaining jejunum. A segmental resection with a one-layered anastomosis was performed using a method described by Freeman (2019) [8]. This kind of anastomosis minimizes luminal diameter reduction [8], as could be evaluated during the re-laparotomy.

Although there was a mild secondary impaction palpated during the initial surgery, the decision was made not to empty and flush the colon through a pelvic flexure enterotomy at that time. It was assessed that this impaction could be resolved on its own, based on the large amount of fluid decompressed from the small intestines into the cecum; also, this decision helped minimize surgery time. However, in the first 36 h after surgery a larger and firmer impaction developed, probably in part secondary to ileus causing fluid retention in the small intestinal lumen. A re-laparotomy was performed to resolve the impaction and from that point the horse recovered well.

Small intestinal strangulations carry a poor to guarded prognosis, with highest mortality in the peri-operative period [22]. Short-term survival rates of horses that are allowed to recover from small intestinal surgery range from 68% and above [8, 22, 28]. Ileus, post-operative colic and repeat laparotomy are factors associated with non-survival after small intestinal resection [8, 29]. Approximately half of the Meckel’s diverticula cases described were euthanized [1, 2, 13, 17, 18, 24, 25, 30]. The horse described here recovered well after repeat laparotomy and was gradually reintroduced to its previous level of work.

In conclusion, Meckel’s diverticula are a rare cause of small intestinal strangulation encountered in horses of a wide age range. In literature, they are often defined as an anomaly of the vitelline duct, presenting as a full-thickness diverticulum on the antimesenteric border of the distal aspect of the jejunum or proximal aspect of the ileum [11]. This case adds to the series of anecdotal reports of anomalies with regard to Meckel’s diverticula in the horse. To our knowledge, this report describes the first mesenteric variant of a Meckel’s diverticulum in a horse with a Y-shaped bifurcation of the distal jejunum. It is worthwhile to report on the different types of aberrations encountered as this may aid accessibility of epidemiological data in future and may help surgeons to identify and recognize these anomalies.

## Data Availability

All supporting data is included in the main paper.

## Abbreviations

CRT: Capillary refill time
PCV: Packed cell volume
WBC: White bloodcell count

## References

[1] Barakzai SZ, Swain JM, Else RW, Licka T, Dixon PM (2003). Two cases of small intestinal strangulation involving Meckel’s diverticulae. Equine Vet Educ.

[2] Verwilghen D, van Galen G, Busoni V, Cassart D, Salciccia A, Sertyn D (2010). Meckels diverticulum as a cause of colic: two cases with different morphological features. Tijdschr Diergeneeskd.

[3] Sprinkle FP, Swerczek TW, Crowe MW (1984). Meckel’s diverticulum in the horse. J. Equine vet. Sci..

[4] Hooper RN (1989). Small intestinal strangulation caused by Meckel's diverticulum in a horse. J Am Vet Med Assoc.

[5] Elsayes KM, Menias CO, Harvin HJ, Francis IR (2007). Imaging manifestations of Meckel's diverticulum. Am J Roentgenol.

[6] De Bosschere H, Simoens P, Ducatelle R, Picavet T (1999). Persistent vitelline arteries in a foal. Equine Vet J.

[7] Jones R, Smith RKW, Mitchell E, Patterson-Kane JC (2004). Persistent vitelline duct cyst in a pony. Equine Vet Educ Education.

[8] Freeman DE, Auer JA, Stick JA, Kümmerle JM, Prang T (2019). Chapter 35: jejunum and ileum. Equine surgery.

[9] Southwood LL (2008). Gastrointestinal tract diverticula: what, when and why?. Equine Vet Educ.

[10] Dearo ACO, de Moraes Marcondes G, Araújo JCO, Grumadas CES, Marino PC, Teixeira WT (2014). Strangulation of the small intestine by an anomalous congenital band in a yearling. Equine Vet Educ.

[11] Hassel DM (2014). Management of abdominal strangulations by mesenteric bands. Equine Vet Educ.

[12] Sarioglu-Buke A, Corduk N, Koltuksuz U, Karabul M, Savran B, Bagci S (2008). An uncommon variant of Meckel’s diverticulum. Can J Surg.

[13] Bartmann CP, Freeman DE, Glitz F, von Oppen T, Lorber KJ, Bubeck K (2002). Diagnosis and surgical Management of Colic in the foal: literature review and a retrospective study. Clin Tech Equine Pract.

[14] Yovich JV, Horney FD (1983). Congenital jejunal diverticulum in a foal. J Am Vet Med Assoc.

[15] Segal SD, Albrecht DS, Belland KM, Elster E (2004). A rare mesenteric location of Meckel's diverticulum, a forgotten entity: a case study aboard USS kitty hawk. Am Surg.

[16] Levack MM, Fiedler AG, Kaafarani H, King DR (2018). Perforation of a mesenteric Meckel’s diverticulum. J Surg Case Rep.

[17] Wefel S, Mendez-Angulo JL, Ernst NS (2011). Small intestinal strangulation caused by a mesodiverticular band and diverticulum on the mesenteric border of the small intestine in a horse. Can Vet J.

[18] Kleinpeter A (2007). Umbilical hernia as Littré's hernia in the foal. History, nomenclature and case report. Tierarztl Prax.

[19] Mahne AT, Janse van Rensburg D, Hewetson M (2017). Ileal hypertrophy and associated true diverticulum as a cause of colic in a horse. J S Afr Vet Assoc.

[20] Abutarbush SM, Shoemaker RW, Bailey JV (2003). Strangulation of the small intestines by a mesodiverticular band in 3 adult horses. Can Vet J.

[21] Freeman DE, Koch DB, Boles CL (1979). Mesodiverticular bands as a cause of small intestinal strangulation and volvulus in the horse. J Am Vet Med Assoc.

[22] van den Boom R, van der Velden MA (2001). Surgery: short- and long-term evaluation of surgical treatment of strangulating obstructions of the small intestine in horses: a review of 224 cases. Vet Q.

[23] Edwards GB (2004). Congenital abnormalities of the equine gastrointestinal tract. Equine Vet Educ.

[24] Röcken M, Reckels FJ, Schmidt-Oechtering GU, Schulte-Ringel AL (1989). (1989) an unusual form of Meckel's diverticulum in the jejunum of a horse. Pferdeheilkunde.

[25] Grant BD, Tennant B (1973). Volvulus associated with Meckel's diverticulum in the horse. J Am Vet Med Assoc.

[26] Hillyer MH (2004). Congenital intestinal abnormalities in the horse. Equine Vet Educ.

[27] Blouhos K, Boulas KA, Tsalis K, Barettas N, Paraskeva A, Kariotis I (2018). Meckel’s diverticulum in adults: surgical concerns. Front Surg.

[28] Freeman DE, Hammock P, Baker GJ, Goetz T, Foreman JH, Schaeffer DJ (2000). Short- and long-term survival and prevalence of postoperative ileus after small intestinal surgery in the horse. Equine Vet J.

[29] Proudman CJ, Edwards GB, Barnes J, French NP (2005). Factors affecting long-term survival of horses recovering from surgery of the small intestine. Equine Vet J.

[30] Viscardi V, Stratievsky GC, Sato HO, Faleiros RR, Alves GES (2009). Strangulation of the small intestine by Meckel's diverticulum and mesodiverticular band in a pregnant mare - case report. Revista Brasileira de Ciência Veterinária.

